# Tamjanika, a Balkan native variety of *Vitis vinifera* L.: Chemical characterization, antibacterial, and anti‐dermatomycosis potential of seed oil

**DOI:** 10.1002/fsn3.2777

**Published:** 2022-02-11

**Authors:** Nikoleta Đorđevski, Dejan Stojković, Jelena Živković, Dejan Pljevljakušić, Elizabeta Ristanović, Biljana Nikolić, Ana Ćirić

**Affiliations:** ^1^ Laboratory of Immunology Institute of Microbiology Medical Military Academy Belgrade Serbia; ^2^ Department of Plant Physiology Institute for Biological Research “Siniša Stanković”—National Institute of Republic of Serbia University of Belgrade Belgrade Serbia; ^3^ Institute for Medicinal Plant Research “Dr Josif Pancic” Belgrade Serbia; ^4^ Faculty of Biology Belgrade University of Belgrade Belgrade Serbia

**Keywords:** antimicrobials, chemical characterization, dermatomycetes, edible oil, functional food, Tamjanika variety, *Vitis vinifera* L.

## Abstract

This study was designed to explore functional food properties of edible seed oil obtained from Tamjanika seeds—autochthonous grape variety of Balkan Peninsula. In order to accomplish our goals, seed oil was isolated by Soxhlet apparatus and chemically characterized regarding fatty acids, carotenoids, tocopherols, and tocotrienols. Antimicrobial activity of the isolated oil was tested by microdilution method. For that purposes, six bacterial species were used, belonging to human infectious agents and food contaminants. Furthermore, the activity of the oil was investigated against clinical isolates of dermatomycetes. Our study has shown that oil of *Vitis vinifera* L. Tamjanika variety was an abundant source of polyunsaturated fatty acids (81.43%) with predominant linoleic acid. HPLC analysis revealed the presence of carotenoid lutein (0.15 mg/100 g). The seed oil was rich in tocotrienols (85.04 mg/100 g) predominating over tocopherols (8.37 mg/100 g). The oil possessed microbicidal activity against all the tested microbes. Bacteria were more sensitive to the effect of the oil (minimum inhibitory concentration [MIC] 7.7–15.4) when compared with oil effect on tested dermatomycetes (MIC 20–40). Our investigation has shown for the first time that grape oil could be active against wide spectrum of bacteria and clinically isolated dermatomycetes. The significance of this study lies in the fact that it pointed out the functional food properties of grape seed oil that was fully chemically characterized.

## Graphical abstract

This study was designed to explore functional food properties of edible fatty oil obtained from Tamjanika seeds—autochthonous grape variety of Balkan Peninsula.

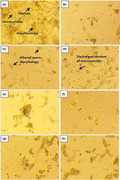

## 1 INTRODUCTION

During the beginning of 20th century, scientific research was focused on the discovery of vitamins and their roles in disease prevention. In time, analytical chemistry achievements enabled identification of various bioactive molecules in natural botanical sources, making their use advanced. The daily intake of certain type of foods could not just strengthen the immune system and lower the risks of infectious disease, but also could prevent development of some chronic diseases. These findings led to the formulation of novel trends in food science and technology describing the functional food concept (Ferreira et al., 2016).

Issaoui and Delgado (2019) gave the report of edible vegetable oils according to Codex Alimentarius Commission (CODEX STAN 210–1999), describing oils such as olive, coconut, palm, sunflower, soybean, and grape suitable for human consumption. Therapeutic properties of grape seed oil have been discussed in written documents from the beginning of the XIV century. This oil was prescribed by an Arab doctor to the King of Castile and Leon to treat skin problems. The king Ferdinand IV protected composition of the oil and labeled it as “oil of the throne” or “royal oil” (Martin et al., 2020). Grape is one of the oldest types of fruits that have been grown since antiquity. Grapefruits are considered as economically very important since they are rich source of sugars, organic acids, vitamins, bioactive compounds, and minerals. It was shown that many grape ingredients, hydrophilic and lipophilic compounds, have diverse positive effects on human health (Nassiri‐Asl & Hosseinzadeh, 2016). Among these hydrophilic compounds, stilbenes found in grape juice, stems, fruits, and seeds have proven as very active with anti‐aging, cardioprotective, and anti‐cancer effects (Pugajeva et al., 2018). Grape seeds contain a high proportion of biologically active lipophilic compounds as well (Górnaś et al., 2019). Various studies have found that grape seeds contain oil in approximately 8%–15% (w/w), especially rich in unsaturated fatty acids (UFAs), such as linoleic acid (72%–76%, w/w) (Ustun Argon et al., 2020).

The grape seed oil is wildly used in European cuisine, especially in Germany, France, and Italy, for its pleasant aroma and taste (Shinagawa et al., 2015). In vitro studies have proven health beneficial effects of grape seed oil regarding anti‐inflammatory, anticancer, antimicrobial, and cardioprotective effects, being previously attributed to the presence of tocopherols, organic acids, carotenoids, and phytosterols (Garavaglia et al., 2016).

Bacterial and fungal infections are currently treated with many available synthetic drugs. Food contaminant and human pathogenic bacteria currently present an important issue for the science that needs to be resolved. Dermatomycosis is a skin infectious disease caused by certain species of dermatomycetes and its treatment is challenging. During the last few decades, there is an emerging problem regarding microbial resistance to antibiotics and antifungals used in current therapeutic practice. This issue arose from improper and overuse of antimicrobial drugs (Cassini et al., 2019). Colossal structural diversity and biological potential of natural products are incomparable with any reference libraries of synthetic drugs (Stojković, Dias, et al., 2020). Recently, there has been an increasing interest for the use of natural matrices to combat microbial pathogenesis. Plants, fruits, vegetables, and their products are shown to be excellent sources of compounds with antimicrobial potential (Ferreira et al., 2016).

Since ancient ethnopharmacological knowledge on grape seed oil pointed to the oil use in skin ailments treatment (Martin et al., 2020), our aim was to isolate oil from the seeds of Tamjanika—autochthonous variety of *Vitis vinifera* (Vitaceae), and to further explore its effect on dermatitis causing fungi and other relevant bacteria for human health. Furthermore, the seed oil was characterized regarding the content of fatty acids, carotenoids, tocopherols, and tocotrienols.

## 2 MATERIALS AND METHODS

### 2.1 Seed material collection and oil extraction

Seeds of grape variety Tamjanika were collected from Topola, Central Serbia during the autumn 2018. Oil from the grounded seeds (15 g) was obtained by Soxhlet extraction with *n*‐hexane, at 60°C for 6 h. After evaporation of the solvent on rotary vacuum evaporator IKA RV 05 (Staufen), grape seed oil was stored in the dark at −25°C.

### 2.2 Chemical Analysis

#### 2.2.1 Chemical characterization of fatty acids

Prior to gas chromatography, fatty acids were transformed to fatty acid methyl esters (FAME) according to standard sulfuric acid procedure (AOAC International). Two hundred microliters of oil was dissolved in 60 ml of benzene/MeOH (1:3) with addition of H_2_SO_4_ and mixture was heated in a water bath at 100°C for 2.5 h. After cooling, it was transferred to a 250 ml separating funnel and 100 ml of H_2_O was added. The extraction was repeated twice with 50 ml of hexane and the combined extracts were rinsed continuously with 20 ml of H_2_O until complete removal of total residual acid (methyl red was used as pH indicator). After drying the solution over anhydrous Na_2_SO_4_, the solvent was removed using IKA RV 05 rotary vacuum evaporator (Staufen). The obtained FAMEs were dissolved in 1 ml of *n*‐hexane and analyzed on the same day (Živković et al., 2017).

GC/flame ionization detector (FID) analysis of obtained FAME was performed on an Agilent Technologies model 7890A gas chromatograph, equipped with split–splitless injector and automatic liquid sampler (ALS), attached to HP‐5MS column (30 m × 0.25 mm, 0.25 µm film thickness) and fitted to FID according to the previously published method (Živković et al., 2017). Carrier gas flow rate (H_2_) was 1 ml/min, injector temperature was 250°C, detector temperature 300°C, while column temperature was linearly programmed to increase from 40 to 260°C (at the rate of 4°C/min), and retained isothermally at 260°C during the next 15 min. Solution of tested sample in *n*‐hexane (15 μl/ml) was consecutively injected by ALS (2 µl, split mode 1:30). Area percent reports, achieved as result of standard processing of chromatograms, were applied as base for the quantification purposes.

The same chromatographic conditions were utilized for GC/MS analysis, using HP G 1800C Series II GCD system (Hewlett‐Packard). Helium was used as carrier gas. Transfer line was heated at 260°C. Mass spectra were acquired in EI mode (70 eV), in the range of 40–450 Da. Sample solutions were injected by ALS (2 µl, split mode 1:30). The constituents were identified by comparison of their mass spectra with those from Wiley275 and NIST/NBS libraries, using various search engines (PBM and NIST). Furthermore, the experimental values for retention indices were determined through the use of calibrated Automated Mass Spectral Deconvolution and Identification System software, (AMDIS ver. 2.64.), compared to those from available literature, and used as additional tool to approve MS findings.

#### 2.2.2 Chemical characterization of carotenoids and tocols

For evaluation of carotenoids and tocols, 100 mg of oil sample was diluted in 1 ml with methanol/tetrahydrofuran (1/1, *v/v*). After centrifugation (5 min, 12,550 *g*), the sample was used for carotenoids analysis. For the analysis of the tocols, 50 μl of the diluted sample was evaporated under a nitrogen stream and redissolved in 1 ml *n*‐hexane/methyl tert‐butyl ether (98 + 2, *v/m*). After centrifugation (5 min, 12,550 *g*), the sample was used for tocols analysis (Živković et al., 2017).

Carotenoid compounds in oil were determined using reversed‐phase HPLC with diode array detector at 450 nm (Merck Hitachi) as reported previously (Bauerfeind et al., 2014; Živković et al., 2017). The chromatographic separation was conducted on a Develosil^®^ RPAQUEOUS C30 column (250 × 4.6 mm i.d., 5 µm particle size) (Phenomenex). The mobile phase included solvent A (methanol) and solvent B (methyl *tert*‐butyl ether). Following gradient elution scheme was applied for separation: 10%–50% B 0–40 min; 50%–60% B 40–42 min; 60% B 42–65 min; 60%–10% B 65–70 min; 10% B 70–75 min. The column temperature was maintained at 13 ± 1°C, while flow was settled to 1 ml/min, and the detection wavelength at 450 nm. Quantification of carotenoids was performed on the basis of comparison of peak areas and their specific absorption maxima with those acquired for external standards with established concentrations considering the recovery of the internal standard (*β*‐apo‐8′‐carotinal). The concentrations of the stock solutions were verified sporadically and were determined from the specific extinction coefficients (Müller et al., 2011; Živković et al., 2017). All experiments were performed in triplicate.

Tocopherols and tocotrienols were determined using a normal‐phase HPLC system with a fluorescence detector (excitation: 292 nm, emission: 330 nm) (Merk‐Hitachi) following previously published method (Kschonsek et al., 2016). An Eurospher 100 Diol analytical column (250 × 4 mm i.d., 7 µm particle size) (Knauer) was applied as stationary phase while *n*‐hexane/methyl *tert*‐butyl ether (98 + 2, v/m) was employed as mobile phase with a flow rate of 1.5 ml/min. Column temperature was maintained at 30 ± 1°C during isocratic analysis. Quantification of tocols was carried out based on comparison of peak areas with those acquired for external standards with well‐known concentrations taking into account the recovery of the internal standard (*α*‐tocopherol acetate). Stock solutions of each standard (tocopherol and tocotrienol) were prepared in ethanol and stored at −30°C. The exact concentrations of the stock solutions were determined spectrophotometrically at their specific absorption maxima (Kschonsek et al., 2016).

### 2.3 Antimicrobial activity

#### 2.3.1 Antibacterial activity

Antibacterial activity of grape seed oil of *V. vinifera*, Tamjanika variety, was evaluated against the following Gram‐positive bacteria: *Staphylococcus aureus* (ATCC 11632), *Micrococcus flavus* (ATCC 10240), *Bacillus cereus* (Clinical isolate); and Gram‐negative bacteria: *Pseudomonas aeruginosa* (PAO1), *Escherichia coli* (ATCC 25922), and *Enterobacter cloacae* (ATCC 35030). Serial dilution technique was employed in 96‐well microliter plates to determine minimum inhibitory concentrations (MICs) and minimum bactericidal concentrations (MBCs) of the seed oil, according to the previous protocols (Patel et al., 2016; Stojković, Drakulić, et al., 2020).

#### 2.3.2 Identification of dermatomycetes

To test the antifungal potential of the seed oil, 11 strains of dermatomycetes were used. Clinical isolates were obtained from the Institute for Skin and Venereal Diseases, Belgrade, Serbia. All the samples were of human origin. Skin swabs of infected patients were inoculated on Dermatophyte test medium (DTM, HiMedia). Pure cultures were isolated and identified by standard microbiological techniques at the Institute for Biological Research "Siniša Stanković"—National Institute of Republic of Serbia, University of Belgrade. The isolates were identified by morphology of colonies on Sabouraud dextrose agar (SDA; Torlak), and microscopically by employing morphometric analyses.

#### 2.3.3 Antifungal activity

A modified microdiluting method (96 system) (Stojković, Dias, et al., 2020) was used to examine potential antifungal activity of the oil. Spores of dermatomycetes were washed from 21‐day‐old cultures, using a sterile 0.85% physiological solution containing 0.1% Tween 80 (v/v). The concentration of the spore suspension was set with a sterile physiological solution to a 1.0 × 10^5^ ml^−1^. Grape seed oil was dissolved in 30% ethanol and its appropriate concentrations (serial two‐fold dilutions) were added in Sabouraud dextrose broth (SDB, Torlak). The plates were incubated for 5 days at 25°C.

The lowest concentrations without visible growth under the microscope were defined as MICs. Minimal fungicidal concentrations (MFCs) were determined by serial reinoculation of 10 µl from the wells without growth into 100 µl of SDA, and re‐incubation for 72 h at 25°C. Estimation of the growth level/absence of growth has been performed by comparison of treated wells with the control ones, that is, the wells in which dermatomycetes grew under the same conditions, but without the test compound (seed oil). The lowest concentration without visible growth is defined as MFC, and it indicated that 99.5% of the initial inoculum was killed.

## 3 RESULTS AND DISCUSSION

### 3.1 Chemical composition of Tamjanika seed oil

As reviewed by Martin et al. (2020), three groups of molecules are predominant in the total lipophilic content of grape seed oil. Fatty acids (FAs) are the most abundant; tocols were also present, while the phytosterol content is the lowest.

Grape seed oil was qualitatively and quantitatively analyzed by advanced chromatographic techniques. It was established that the oil was constituted of 14 fatty acids (Table 1). The percentage of saturated and UFAs in the oil of Tamjanika grape seed was as follows: saturated fatty acids (SFAs): 11.04%; mono‐unsaturated fatty acids (MUFAs): 7.48%; polyunsaturated fatty acids (PUFAs): 81.43% (Table 1). The percentage of UFAs, according to the position of the double bond, was as follows: *ω*‐3:0.20%; *ω*‐6:81.23%; *ω*‐7:0.16%; *ω*‐9:7.32%.

**TABLE 1:** Fatty acids composition in Tamjanika seed oil

Compound		% of total fatty acids
Myristic acid	C14:0	0.05
Palmitoleic acid	C16:1, cis‐9	0.16
Palmitic acid	C16:0	10.30
Margaric acid	C17:0	0.08
Linoleic acid	C18:2	81.09
Oleic acid	C18:1	6.85
Stearic acid	C18:0	0.02
11,14‐Eicosadienoic acid	C20:2	0.02
γ‐Linolenic	C18:3	0.12
α‐Linolenic acid	C18:3	0.20
Gondoic acid	C20:1	0.47
Arachidic acid	C20:0	0.27
Heneicosanoic acid	C21:0	0.28
Behenic acid	C22:0	0.04
Total SFA		11.04
Total MUFA		7.48
Total PUFA		81.43

Abbrevaitions: MUFA, mono‐unsaturated fatty acid; PUFA, polyunsaturated fatty acid; SFA, saturated fatty acid.

The most abundant fatty acid in the tested oil was linolenic acid (81.09%). Our results are in accordance with previous studies reporting linoleic acid as one of the most dominant fatty acids in the grape seed oil (Fernandes et al., 2013; Garavaglia et al., 2016; Lachman et al., 2015; Lutterodt et al., 2011; Mahanna et al., 2019; Sabir et al., 2012). Linoleic acid was found as the most prevalent fatty acid in the seed oils obtained from 19 interspecific hybrids of *Vitis* species studied by Górnaś et al. (2019), with ‘Hasanskij Sladkij’ (*V. vinifera* × *V*. *amurensis*), ‘Liepajas Dzintars’, ‘Mali’, ‘V4‐5‐2’ (*V. vinifera* × *V*. *amurensis* × *V*. *riparia*) containing more than 80% of linoleic acid of the total fatty acids. The second most dominant fatty acid in our sample was palmitic acid. This acid was found as the second most dominant in the seed oils of several *Vitis* hybrids (*V. vinifera* × *V*. *amurensis* × *V*. *riparia*) (Górnaś et al., 2019). Further, previous studies have shown that UFAs comprise almost 90% of the total fatty acid composition (Fernandes et al., 2013; Garavaglia et al., 2016; Lachman et al., 2015; Lutterodt et al., 2011; Sabir et al., 2012); our study indicated that total UFAs amounted 88.91% of the total fatty acids, making our results consistent with the previous reports.

Linoleic acid belongs to the group of PUFAs. It is the most abundant fatty acid in nature and is a precursor of other *ω*‐6 fatty acids. Omega‐3 fatty acids are synthesized from α‐linoleic acid (Nagy & Tiuca, 2017). Over the years, it has been found that *ω*‐3 fatty acids have a great impact on human health, especially playing a major role in the prevention of cardiovascular disease (Nagy & Tiuca, 2017). Linoleic acid also has significant antimicrobial potential (Huang et al., 2010). Short‐chain fatty acids have an effect on the prevention of diabetes and obesity, colon cancer, and have an antimicrobial effect. Medium‐chain fatty acids are involved in the prevention of obesity. In addition, long‐chain fatty acids, which are the most abundant in this oil, have various positive effects on several human diseases such as: rheumatoid arthritis, Alzheimer's disease, type II diabetes, and also participate in the prevention of cardiovascular diseases and obesity (Nagy & Tiuca, 2017).

Tocols (tocopherols and tocotrienols) are monophenols derived from 6‐hydroxy‐2‐methyl‐2‐phytylchroman, and have many practical applications in food and pharmaceutical industries (Durazzo et al., 2021). Tocopherols and tocotrienols are soluble in fats, which means that our body deposits it and can use it when needed. There are reports on different functional features of tocols, including antidiabetic, cardioprotective, antiobesity, and anticancer effects. Moreover, the functions of tocotrienols and tocopherols are different, and a recent study indicated a more effective activity of the tocotrienols than that of the *α*‐tocopherol (Durazzo et al., 2021). There are reports that tocols protect against oxidative damage in various cell types, such as: neurons, fibroblasts, osteoblasts, chondrocytes, muscle cells, and tooth pulp cells. *α*‐Tocopherol also plays an important role as an antioxidant molecule, possessing also an anti‐inflammatory effect (Rhouma et al., 2013). Our results showed that seed oil was rich in tocopherols (8.37 mg/100 g oil) and tocotrienols (85.04 mg/100 g oil) (Table 2). Similar observations were reported in 19 interspecific grape hybrids, where the total concentration of tocopherol and tocotrienol homologues was between 0.785 and 9.033 g/kg oil. The concentrations of *α*‐tocopherol, *γ*‐tocopherol, and *γ*‐tocotrienol in grape seeds obtained in the present study were comparable with the results for the oils recovered from the seeds of *V. vinifera* × *V*. *amurensis* × *V*. *riparia* (Górnaś et al., 2019).

**TABLE 2:** Composition of tocopherols (T) and tocotrienols (T_3_) in Tamjanika oil, presented in mg/100 g oil (mean ± *SD*)

Sample	*α*–T	*β*–T	*γ*–T	*δ*–T	*α*–T_3_	*β*–T_3_	*γ‐*T_3_	Σ Tocopherols	Σ Tocotrienols
Tamjanika seed oil	3.11 ± 0.09	nd	0.49 ± 0.06	nd	12.47 ± 0.44	nd	23.74 ± 0.71	8.37 ± 0.33	85.04 ± 3.75

The most biologically active form of tocopherols is *α*‐tocopherol, which was the most abundant in this oil (3.11 mg/100 g, Table 2). It was previously shown that liposoluble *α*‐tocopherol can fight against antibiotic resistance caused by bacterial lipocalins (Georgousopoulou et al., 2017).

Tocotrienols were dominating over tocopherols for more than ten times in our sample, with *γ*‐tocotrienol being the most common (23.74 mg/100 g ili µmol/100 g, Table 2). As reviewed by Ustun Argon et al. (2020), the forms of *γ*‐tocotrienol and *α*‐tocotrienol are the most dominant tocotrienol and these two components have the highest variations between grape varieties. These compounds have shown potential positive effects on human health, especially on cognitive abilities (Prasad, 2011), heart (Meganathan & Fu, 2016), and diabetes (Britton & Khachik, 2009).

Regarding carotenoids, their total content was 0.27 mg/100 g in our sample. Among tested carotenoids, only lutein (0.15 mg/100 g oil) was detected and measured (Table 3). Lutein has positive effects on eyes: reduces the percentage of macular degeneration caused by age and reduces the risk of developing cataracts (Chew et al., 2013). Studies about the carotenoid content of grape seed oil are very limited. The grape seed oils from Brazilian market contained β‐carotene, while the Polish commercial oils contained no carotenoid compounds (Ustun Argon et al., 2020). In recent years, it has been found that carotenoids can also contribute to reducing the risk of developing various chronic diseases, including several types of cancer, cardiovascular disease, and skin and eye disorders (Krinsky & Johnson, 2005; Rodriguez‐Concepcion et al., 2018).

**TABLE 3:** Carotenoids composition in Tamjanika oil presented in mg/100 g oil (mean ± *SD*)

Sample	Lutein	Zeaxanthin	(E)‐*β*‐Carotin	(9Z)‐*β*‐Carotin	(13Z)*‐β*‐Carotin	∑ Carotenoids
Fatty oil	0.15 ± 0.004	nd	nd	nd	nd	0.27 ± 0.01

### 3.2 Antimicrobial activities of Tamjanika grape seed oil

Food contaminants and enteropathogenic bacteria were present among the tested strains. All of the investigated bacteria could also infect skin in immunocompromised patients, making them suitable targets for our study. The results of the antibacterial activity of the grape seed oil are shown in Table 4. The values of MIC and MBC are expressed in mg/ml. The obtained results showed that seed oil was an antibacterial agent, with the MICs and MBCs in the ranges 7.7–15.4 mg/ml, and 15.4–30.8 mg/ml, respectively. *M. flavus* and *E. coli* were shown to be the most resistant, with a MIC of 15.4 mg/ml and MBC of 30.8 mg/ml. All other bacteria showed the same, somewhat higher sensitivity to the oil (MIC and MBC were 7.7 and 15.4 mg/ml, respectively). The antimicrobial activity of grape seed oil has been reported against certain pathogens, such as *S. aureus* and *E. coli* (Rotava et al., 2009), making our results supported by these findings.

**TABLE 4:** Antibacterial effects of Tamjanika seed oil

Bacteria	MIC (mg/ml)	MBC (mg/ml)
*Bacillus cereus*	7.7	15.4
*Escherichia coli*	15.4	30.8
*Pseudomonas aeruginosa*	7.7	15.4
*Enterobacter cloacae*	7.7	15.4
*Staphylococcus aureus*	7.7	15.4
*Micrococcus flavus*	15.4	30.8

Abbreviations: MBC, minimum bactericidal concentration; MIC, minimum inhibitory concentration.

**FIGURE 1:**
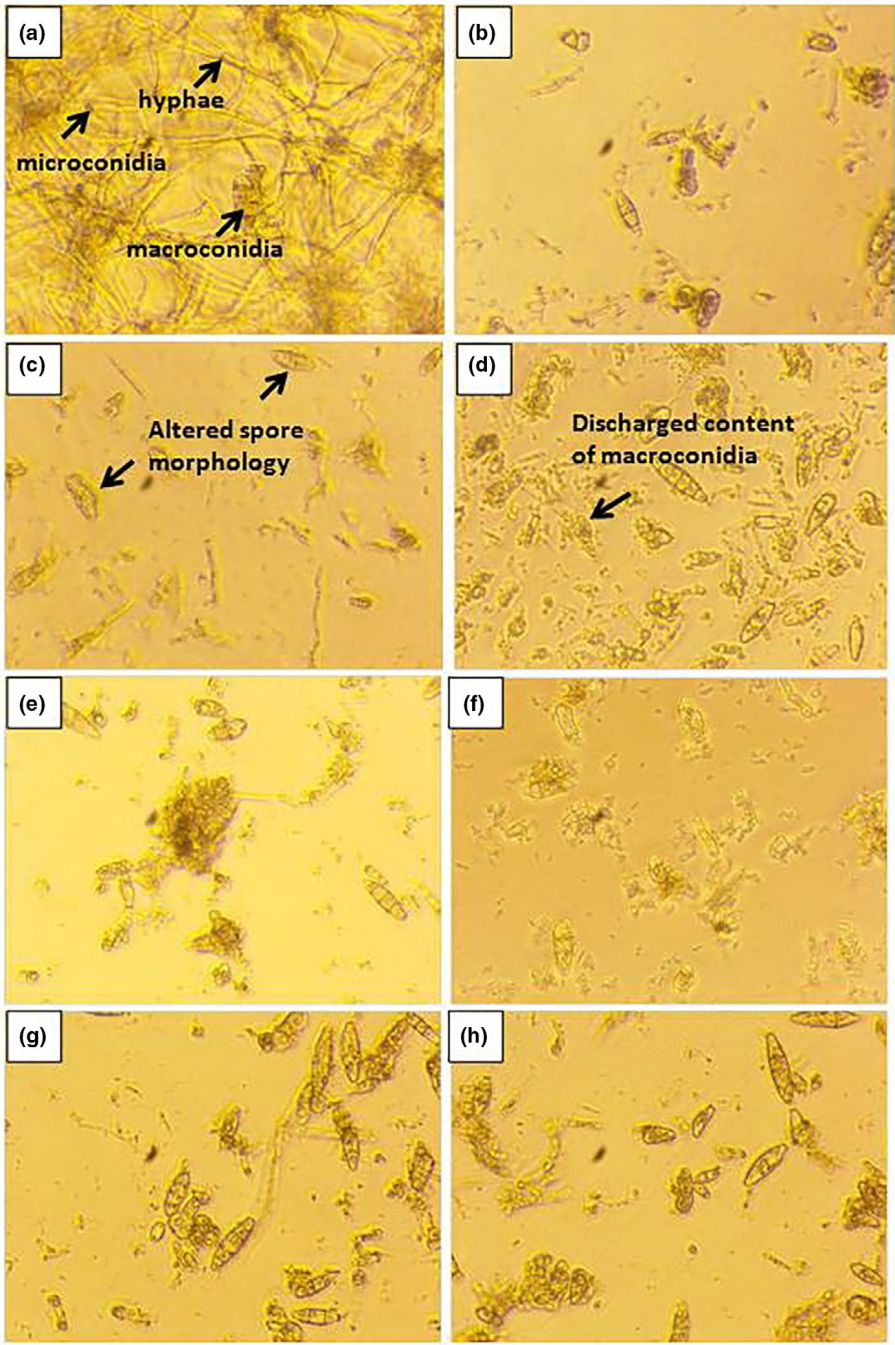
Antifungal activity of Tamjanika seed oil different concentrations (b–h) on *Microsporum canis*, visualized by inverted light microscopy (magnification 40×). (a) *M. canis—*Control, (b) 0.32 mg/ml, (c) 0.63 mg/ml, (d) 1.25 mg/ml, (e) 2.5 mg/ml, (f) 5 mg/ml, (g) 10 mg/ml, (h) 20 mg/ml

The fact that this research deals with the antifungal effect against dermatomycetes is especially valuable, because dermatomycoses are particularly difficult to treat and have great potential to cause recurrent infections (de Macedo & Freitas, 2020). Isolated fungi were identified as *Trichophyton mentagrophytes* (3 isolates), *Trichophyton rubrum* (2 isolates), *Trichophyton verrucosum* (1 isolate), *Microsporum gypseum* (3 isolates), and *Microsporum canis* (2 isolates). They were used for antifungal susceptibility assay. Based on the results, it could be noted that the oil had some effect on all tested dermatomycetes. Although MICs and MFCs ranged from 20 to 40 mg/ml, obtained results can be considered as important, due to high resistance of dermatomycetes to conventionally applied fungicides (Martinez‐Rossi et al., 2018). All dermatomycetes showed similar sensitivity to this oil with MFC of 40 mg/ml, except one isolate of *M. canis* which was the most sensitive fungus with MIC and MFC determined at 20 mg/ml (Table 5). Figure 1a–h illustrates how the most sensitive isolate of *M. canis* reacted to different concentrations (0.32, 0.63, 1.25, 2.5, 5, 10, 20, 40, 80, and 160 mg/ml) of grape seed oil. Figure 1a shows the control of *M. canis*, in a well of microdilution plate without added grape seed oil. A multitude of hyphae, micro‐, and macroconidia could be seen. Figure 1b shows how the oil acted at an added concentration of 0.32 mg/ml. Micro‐ and macroconidia were clearly visible, but the number of hyphae was reduced. At a concentration of 0.63 mg/ml (Figure 1c), it could be noticed that the oil begun to affect the morphology of spores, making them deformed. At a concentration of 1.25 mg/ml (Figure 1d), we saw that the macroconidia have ruptured, under the action of the oil, and that their contents have been discharged into the external environment. Going toward increasing concentrations of 2.5, 5, and 10 mg/ml (Figure 1e–g), we have noticed that more and more spores are degenerating, cracking, and releasing their contents. Finally, at a concentration of 20 mg/ml (Figure 1h), we observed only a few spores in total and determined this concentration as MFC (at which 99.5% of the initial inoculum was killed).

**TABLE 5:** Antifungal activity of Tamjanika oil

Fungi	MIC (mg/ml)	MFC (mg/ml)
*Trichophyton mentagrophytes* 1	40	40
*Trichophyton mentagrophytes* 2	40	40
*Trichophyton mentagrophytes* 3	20	40
*Trichophyton rubrum* 1	20	40
*Trichophyton rubrum* 2	20	40
*Trichophyton verrucosum*	40	40
*Microsporum canis* 1	40	40
*Microsporum canis* 2	20	20
*Microsporum gypseum* 1	40	40
*Microsporum gypseum* 2	40	40
*Microsporum gypseum* 3	40	40

Abbreviations: MBC, minimum bactericidal concentration; MFC, minimal fungicidal concentration.

Cold‐pressed oregano (*Origanum vulgare*) oil was rich with linoleic, oleic, stearic, and palmitic acids and possessed a very low minimal lethal concentration against bacteria and dermatomycetes (Assiri et al., 2015). Ramadan et al. (2012) reported that the main fatty acids in cold‐pressed black cumin seed oil were linoleic, oleic, and palmitic acids, while cumin seed oil possessed petroselinic acid and linoleic acid. Black cumin seed oil was more active against bacteria and fungi than cumin seed oil. The antimicrobial potential of the plant oils could target different components of microbial cell, primarily the cell membrane, cytoplasm, and in some cases, they radically change the morphology of cells (Nazzaro et al., 2013).

## 4 CONCLUSIONS

Qualitative and quantitative analysis of fatty acids of the oil derived from seeds of *V. vinifera*, Tamjanika variety was performed, showing the presence of unsaturated (mono‐ and poly‐) and saturated fatty acids, among which polyunsaturated fatty acids were dominating. Fatty acid profile revealed that the most abundant polyunsaturated fatty acid was linoleic acid. HPLC analysis of tocols showed that Tamjanika grape seed oil was rich in tocols. The most common tocols were tocotrienols, with a higher share in the oil, when compared to tocopherols. HPLC analysis of carotenoids showed that the main carotenoid in this oil was lutein. Majority of investigated bacteria showed the same sensitivity to the effect of the oil. The seed oil showed an interesting antifungal potential toward tested dermatomycetes. However, according to our knowledge, this study is the first to explore antibacterial potential and effect on dermatomycetes of grape seed oil obtained from Tamjanika variety, showing the broader spectrum of activity on bacteria and fungi associated with the skin infections. Having in mind that grape seed oil was very rich in biologically active compounds, and that it has shown antibacterial potential, but also some effectiveness against tested dermatomycetes, we recommend further product development based on Tamjanika seed, with attributed novel functional properties described in here.

## CONFLICT OF INTEREST

The authors declare that the research was conducted in the absence of any commercial or financial relationships that could be construed as a potential conflict of interest.

## Data Availability

All data are presented within the manuscript.
